# Intraspecific Variations in Ecomorphological Functional Traits of Montane Stream-Dwelling Frogs Were Driven by Their Microhabitat Conditions

**DOI:** 10.3390/ani15152243

**Published:** 2025-07-30

**Authors:** Xiwen Peng, Da Kang, Guangfeng Chen, Suwen Hu, Zijian Sun, Tian Zhao

**Affiliations:** College of Fisheries, Southwest University, Chongqing 400715, China; pxw@swu.edu.cn (X.P.); kangda1013@126.com (D.K.); 13984402874@139.com (G.C.); taoye574@gmail.com (S.H.); sunzj19@outlook.com (Z.S.)

**Keywords:** functional morphology, phenotypic plasticity, amphibian conservation, microhabitat variables

## Abstract

Understanding how habitat conditions drive morphological adaptations in animals is critical in ecology, yet amphibian studies remain limited. This study investigated intraspecific variation in ecomorphological traits of three montane stream-dwelling frogs (*Quasipaa boulengeri*, *Amolops sinensis*, and *Odorrana margaratae*) across elevation gradients in Tianping Mountain, China. Using morphological measurements and environmental variables collected from ten transects, we analyzed functional traits related to feeding and locomotion and assessed their associations with microhabitat variables. Significant trait differences between low- and high-elevation groups were detected only in *Q. boulengeri*, with high-elevation individuals exhibiting greater body mass and shorter hindlimbs. Redundancy analysis demonstrated that microhabitat variables, particularly air humidity, flow rate, and rock coverage, were linked to trait variations. For example, air humidity and flow rate significantly influenced *Q. boulengeri*’s body and limb proportions, while flow rate affected *A. sinensis*’s snout and limb morphology. In addition, sex and seasonal effects were also associated with trait variations. These results underscore amphibians’ phenotypic plasticity in response to the environment and highlight the role of microhabitat complexity in shaping traits. By linking habitat heterogeneity to eco-morphology, this study advocates for conservation strategies that preserve varied stream environments to support amphibian resilience amid environmental changes.

## Simple Summary

Protecting wildlife requires understanding how animals adapt to their surroundings, yet amphibians in montane streams remain understudied. This research studied three amphibian species in Tianping Mountain, China, to explore how their physical traits, such as body size, leg length, and head shape, respond to varying habitat conditions. By comparing individuals from high- and low-elevation sites, we found that those living in colder, higher-altitude areas developed larger bodies and shorter legs, adaptations that may aid in conserving heat. One species also exhibited broader heads, which could enhance prey capture in fast-flowing streams. These morphological changes highlight the amphibians’ ability to adjust to specific environmental pressures. Our findings underscore the importance of maintaining diverse habitats (e.g., streams with varying water depths and flow rates) to support amphibian populations. Since amphibians serve as indicators of environmental health, protecting their habitats contributes to the well-being of entire ecosystems, including human communities. This study offers practical guidance for conservation efforts, emphasizing the need to preserve habitat heterogeneity to ensure amphibian resilience in the face of climate change and human disturbance.

## 1. Introduction

Ecomorphological functional traits are crucial to understanding how species within the same ecological guild can coexist through niche segregation [1]. These morphological and functional attributes of species are often shaped by biotic interactions and are closely linked to the exploitation of ecological resources. Accordingly, many studies were conducted to quantify the functional traits difference between species (i.e., interspecific variation) and to explore how these traits relate to ecological resource use [2]. For instance, Zhao et al. quantified ecomorphological functional trait differences between native and non-native fish species in gravel pits in southern France, and they suggested that such trait divergence may facilitate their coexistence [3]. Moreover, an increasing number of studies have indicated that functional trait variations within species could be as substantial as interspecific variation, playing a critical role in determining individual fitness and ecological performance [4]. Therefore, exploring intraspecific variation in functional morphology provides valuable insights into population structure and ecological dynamics.

It is well recognized that species’ ecomorphological functional traits can be influenced by external environmental variables due to phenotypic plasticity [2,5]. These traits reflect species’ adaptations to local environments and are closely linked to specific ecological functions. For instance, fish with streamlined body shapes are typically found in fast-flowing waters, while those with round-shaped mouths are often adapted for filter feeding [6]. Therefore, exploring the environmental determinants of species’ ecomorphological functional traits can help us better understand the responses of communities to changes in environmental conditions. Similarly, functional morphological traits within species can also strongly vary. Such intraspecific variation has been widely documented in fish [4], lizards [7], birds [8], and mammals [9]. However, amphibians have received relatively little attention in this context. Indeed, amphibian ecomorphological functional traits are equally important for their performance in ecosystems. Specifically, head morphology is associated with feeding strategies, while body and limb morphology are closely related to locomotion [10,11]. Therefore, intraspecific variation in amphibian functional morphology may reflect differences in ecological niche utilization. More importantly, whether such variation is shaped by habitat conditions remains largely unexplored and warrants further investigation.

Amphibians are the most threatened vertebrate group across the world. According to the IUCN Red List, approximately 41% of amphibian species are threatened with extinction globally [12]. This crisis is particularly pronounced in mountain regions, which are recognized as centers of endemism and home to a high proportion of threatened species [13]. Therefore, quantifying the relationships between intraspecific trait variation and habitat conditions in mountain-dwelling amphibians can provide important insights for conservation strategies. Using three montane stream-dwelling frogs (i.e., *Quasipaa boulengeri*, *Amolops sinensis*, and *Odorrana margaratae*) as the models, the main objective of this study was to assess how microhabitat conditions influence amphibians’ intraspecific variation in functional morphology. Specifically, we (1) compared the functional trait differences within species between different elevational groups; (2) identified the microhabitat variables associated with distinct functional traits across elevations; and (3) investigated the relationships between amphibians’ functional traits and microhabitat characteristics.

## 2. Materials and Methods

### 2.1. Study Area

The present study was conducted in Tianping Mountain, the core area of Badagongshan National Nature Reserves, located in northwestern Hunan Province, China (29°42′51″–29°47′14″ N, 109°54′22″–110°10′15″ E). The region spans an elevational gradient from 300 to 1890 m, covering two distinct climatic zones. Specifically, it is relatively warm (mean annual temperature: 13.7–15.9 °C) in the low-elevation area (300–1000 m), with crops and evergreen broad-leaved forests dominating this area. In contrast, the mean annual temperature is below 10.0 °C in the high-elevation area (1000–1890 m), in which the main vegetation cover is evergreen deciduous broadleaf forests [14].

### 2.2. Data Collection

**Figure 1:**
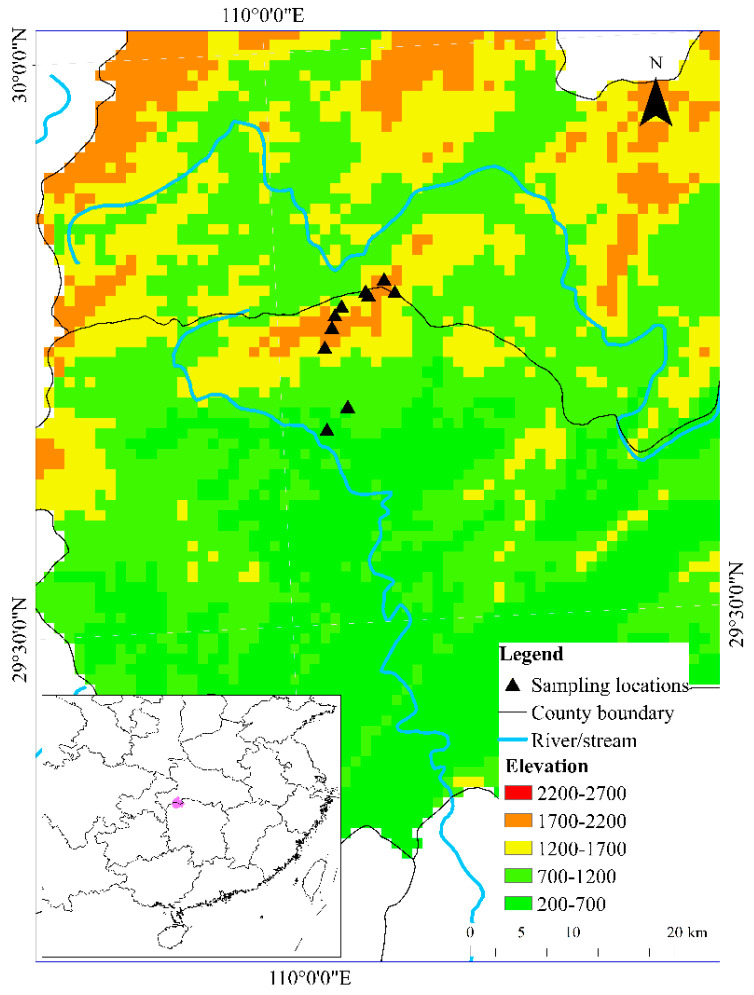
Map of the study area showing Tianping Mountain, China (the triangles denote transects selected in the present study to survey amphibians).

Ten transects (200 m × 2 m) were established along mountain stream tributaries spanning a low-to-high elevational gradient (Figure 1, Table S1). These transects were divided into two groups according to their elevations. Specifically, the five located below 1000 m were classified as the low-elevation group, while the other five above 1000 m were designated as the high-elevation group. To minimize spatial autocorrelation, these transects were separated by mountain ravines or streams, with a minimum distance of 1.5 km between them. Amphibian surveys were conducted in April, June, and August in 2017, separately. These three time periods corresponded to three distinct seasonal periods in this region (i.e., spring, early summer, and mid-summer), covering the main activities of amphibians such as breeding, foraging, and migration. We employed a combination of distance sampling and quadrat sampling under nocturnal time-constrained visual encounter surveys to search for amphibians along the transects. Detailed sampling protocols are provided in our previous work [15]. All adult individuals of the three target species encountered were captured, transported to a nearby dry place for morphological measurements, and subsequently released at their original capture locations.

**Figure 2:**
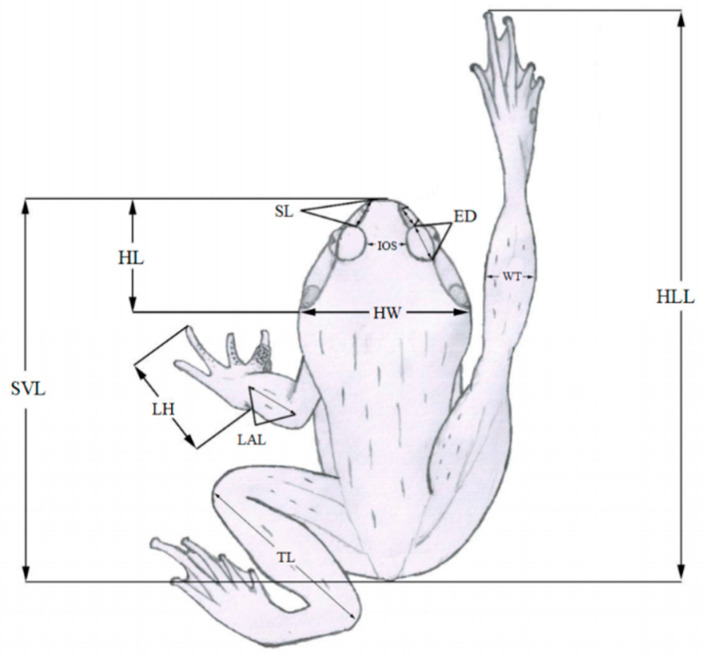
Measurement of amphibians’ external morphology traits. The abbreviated meanings of the letters are follows: SVL, snout-vent length; HL, head length; HW, head width; SL, snout length; ED, diameter of eye; IOS, interorbital space; LAL, length of lower arm; LH, length of hand; HLL, hindlimb length; TL, tibia length; WT, width of tibia.

A set of 11 morphological traits were measured for each individual, including snout-vent length (SVL), head length (HL), head width (HW), snout length (SL), diameter of eye (ED), interorbital space (IOS), length of lower arm (LAL), length of hand (LH), hindlimb length (HLL), tibia length (TL), and width of tibia (WT; Figure 2). These traits were measured directly using a digital caliper to the nearest 0.01 mm. Moreover, body weight of the individuals was recorded using an electronic scale to the nearest 0.01 g. All the measurements were conducted by the same person to ensure consistency. Based on previous published literature [16,17,18], 11 eco-morphological functional traits were obtained through the calculation of all the above morphological traits (Table 1), which can reflect two key ecological functions of amphibians (i.e., food acquisition and locomotion).

We also recorded 16 microhabitat variables in each transect during the sampling events conducted in April, June, and August, separately. These variables were selected based on previous studies indicating their influence on amphibian distribution, activity, and various life history processes [10,19,20,21]. Specifically, air temperature (AT) was measured after sunset (20:00–23:00) at each transect using a mercury thermometer. Air humidity (AH) was recorded with a digital humidity meter (Peakmeter MS6508, Huayi, Guilin, China; 20:00–23:00). Water temperature (Wt), water pH (pH_w), and water conductivity (Wc) were measured by using a portable fluorescence photometer (Orion, Thermo Fisher Scientific, Waltham, MA, USA). Water depth (Wd), water width (Ww), and fallen leaf depth (Fld) were measured by using a steel tape. Soil pH (pH_s) was measured with a soil pH Meter (ZD-05, Zhengda, Taizhou, China). Flow rate (Fr) was recorded using a velocimeter (LS1206B, Xiangruide, Nanjing, China). All the above microhabitat variables were measured at 20 m intervals along each transect, and the averaged values were used for further analyses. Canopy density (Cd) was assessed using a spherical densitometer. The number of trees was manually counted. Rock coverage (Rc; the ratio of rock-covered length to the total transect length), shrub coverage (Sc; the ratio of shrub-covered length to the total transect length), and fallen leaf coverage (Flc; the ratio of fallen leaf coverage length to the total transect length) were estimated by the same person to ensure consistency. We also recorded the elevation (Ele) of each transect by using a GPS (ICEGPS 660, Binhe, Shenzheng, China). Therefore, each transect was associated with one set of environmental variables per season. Further methodological details are available in a previous study [15].

### 2.3. Statistical Analyses

All morphological and environmental variables were standardized using Z-score transformation prior to analyses to eliminate scale differences. Principal Component Analysis (PCA) was first performed on all standardized eco-morphological functional traits to identify the major axes of morphological variation. To evaluate differences in functional morphology between low- and high-elevation groups, permutational multivariate analysis of variance (PERMANOVA) was conducted separately for each species [22]. Given the potential influence of sexual dimorphism and seasonal variation, both sex and season were included as factors in the PERMANOVA analyses. In parallel, PCA was also applied to the microhabitat variables from transects where the target species were recorded, and loadings were used to interpret their contributions to each principal component. Finally, redundancy analysis (RDA) was performed to investigate how specific microhabitat variables explain variation in functional traits [23]. Sex, elevation, and season were incorporated as explanatory variables in the RDA models to account for their potential effects (baseline: high elevation and female). A simplified RDA model was selected using the *tep* function, and variance inflation factors (VIFs) were calculated using the *vif.cca* function to detect multicollinearity among explanatory variables. Variables with VIF values greater than 20 were excluded from the final model. Model performance and the significance of the constrained axes were evaluated via ANOVA permutation tests with 1000 random permutations. Only statistically significant variables were displayed in the RDA plots. All analyses were performed in R [24]. PCA and RDA analyses were conducted using the vegan package [25]. Spearman’s rank correlations were calculated using the psych package [26].

## 3. Results

A total of 202 adults were captured during four surveys. Specifically, we collected 24 *Q. boulengeri*, 40 *A. sinensis*, and 25 individuals of *O. margaratae* in the low-elevational transects and 31 *Q. boulengeri*, 27 *A. sinensis*, and 55 individuals of *O. margaratae* in the high-elevational transects. The mean total lengths of *Q. boulengeri* were 78.13 mm ± 14.11 for males (SD; range: 57.94–107.59 mm) and 97.57 ± 19.80 for females (SD; range: 67.13–150.23 mm). The mean total lengths of *A. sinensis* were 49.21 mm ± 4.86 for males (SD; range: 39.46–59.32 mm) and 56.66 mm ± 4.01 for females (SD; range: 48.36–62.84 mm). The mean total lengths of *O. margaratae* were 76.08 mm ± 8.65 for males (SD; range: 51.88–94.67 mm) and 91.89 ± 5.35 for females (SD; range: 78.42–102.40 mm). The mean body weights of *Q. boulengeri* were 57.90 g ± 30.77 for males (SD; range: 19.60–127.60 g) and 113.22 g ± 72.44 for females (SD; range: 38.83–309.40 g). The mean body weights of *A. sinensis* were 13.61 g ± 7.40 for males (SD; range: 4.90–49.23 g) and 15.28 g ± 3.34 for females (SD; range: 9.10–24.31.40 g). The mean body weights of *O. margaratae* were 40.74 g ± 16.14 for males (SD; range: 12.10–83.23 g) and 65.97 g ± 18.25 for females (SD; range: 24.37–100.30 g).

### 3.1. Intra-Species Functional Morphology Difference vs. Elevation

**Figure 3:**
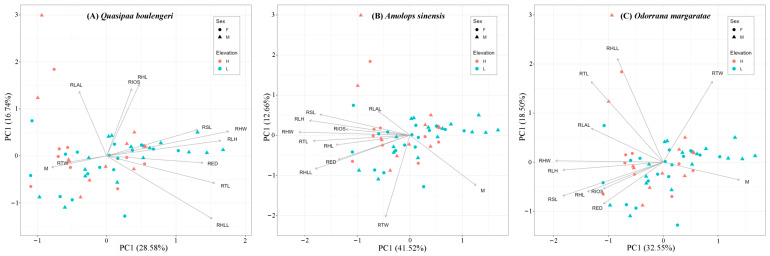
Spatial variations of amphibian functional morphology using a scatter diagram by elevation and sex (L: low-elevational group; H: high-elevational group; F: females; M: males).

Based on the PCAs of standardized ecomorphological functional traits, the first two principal components accounted for 45.32%, 54.18%, and 51.05% of the total trait variation for *Q. Boulengeri*, *A. sinensis*, and *O. margaratae*, respectively. For *Q. Boulengeri*, PC1 was primarily associated with RHW, RLH, RHLL, and RTL, while PC2 was mainly influenced by RHL, RIOS, RLAL, and RHLL. Individuals with higher PC1 scores generally exhibited wider heads and longer hands, hindlimbs, and tibias. In contrast, individuals with higher PC2 scores tended to have longer heads and forelimbs, wider interorbital spaces, and relatively shorter hindlimbs. For *A. sinensis*, PC1 was mainly driven by RHL, RHW, RSL, RLH, RHLL, and RTL, whereas PC2 was predominantly influenced by RTW. Higher PC1 values corresponded to individuals with shorter and narrower heads, as well as shorter snouts, forelimbs, and hindlimbs. Higher PC2 values indicated shorter tibia width. In terms of *O. margaratae*, PC1 was largely explained by RHW, RSL, and RLH, while PC2 was primarily contributed by RHLL, RTL, and RTW. Individuals with increasing PC1 values showed narrower heads and shorter snouts and forelimbs, whereas increasing PC2 values were associated with longer hindlimbs and longer and wider tibias (Table 2). Based on the results of PERMANOVA analyses, functional trait differences were detected between low and high elevational groups for *Q. Boulengeri* (Figure 3; *p* = 0.014). Moreover, this species also exhibited trait differences between sex and seasonal groups (Figure 3; *p* = 0.002 and *p* = 0.010; respectively). For *A. sinensis* and *O. margaratae*, there were no intraspecific trait differences between elevational groups (Figure 3; *p* = 0.220 and *p* = 0.268, respectively). However, trait differences can be observed between sex and seasonal groups (*A. sinensis*: *p* = 0.001 and *p* = 0.001, respectively; *O. margaratae*: *p* = 0.001 and *p* = 0.001, respectively).

### 3.2. The Relationships Between Amphibians’ Functional Traits and Microhabitat Variables

PCA analyses of microhabitat variables revealed that the first two principal components accounted for 67.80% of the total variation for the three target species. Specifically, PC1 was mainly influenced by Ele, AT, Wt, Wd, Ww, Cd, and Fr, while PC2 was primarily driven by Ww, Rc, and Fr (Table 3). With increasing PC1, the microhabitat will exhibit higher elevation, colder temperature, smaller water bodies, slower flow rate, and higher canopy density. In contrast, the microhabitat will contain more rocks, with larger water bodies and higher flow rates. For the distribution of three target species, *Q. Boulengeri* males were associated with lower PC1 values and higher PC2 values, while the females were associated with higher PC1 values and higher PC2 values. *A. sinensis* (both males and females) and *O. margaratae* males were linked to higher PC1 values. In addition, *O. margaratae* females were connected with lower PC1 values (Figure S1).

### 3.3. Different Microhabitat Variables Affect Different Functional Traits

**Figure 4:**
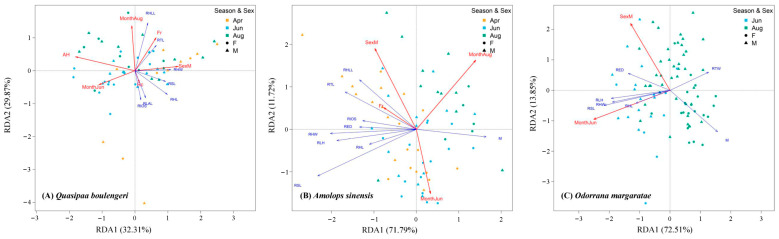
Redundancy analysis triplots showing the relationships between anuran functional traits and microhabitat variables for three target species. The RDA triplot only retains the significant microhabitat variables (*p* < 0.05), which are represented by red lines. Blue lines indicate the functional traits. Circles and triangles indicated females and males, respectively. Different colors represent the individuals encountered in different seasons (April: orange; June: blue; August: green).

The RDA model revealed that the relationships between functional traits and microhabitat variables were significant for all amphibian species (*Q. boulengeri*: *R^2^* = 0.31, *p* = 0.001; *A. sinensis*: *R^2^* = 0.33, *p* = 0.001; and *O. margaratae*: *R^2^* = 0.25, *p* = 0.001). The first two RDA axes explained 32.31% and 29.87% of the explainable variance for *Q. boulengeri*, respectively. Functional traits in this species were significantly influenced by three microhabitat variables (i.e., AH, Fr, and Rc), as well as by sex and season. Specifically, males were significantly and positively correlated with RHLL, RHW, and RTL, whereas individuals sampled in June showed significant negative correlations with these traits. Moreover, RHL, RSL, RLAL, and RIOS were positively associated with RC but negatively associated with AH and the month of August (Figure 4A). For *A. sinensis*, the first two RDA axes explained 71.79% and 11.72% of the explainable variance, respectively. Functional traits were significantly affected by one microhabitat variable, sex, and season. Specifically, Fr and males were positively correlated with RIOS, RTL, and RHLL. However, individuals sampled in June exhibited entirely opposite patterns. Those sampled in August were negatively associated with RSL, RHL, and RLH (Figure 4B). In addition, the first two RDA axes explained 72.51% and 13.85% of the explainable variance for *O. margaratae*, respectively. Specifically, males were significantly and positively associated with RED, but negatively related to M. Individuals sampled in June were significantly and positively associated with RLH, RHW, RSL, and RHL but were negatively correlated with RTW (Figure 4C).

## 4. Discussion

The present study investigated eco-morphological functional trait variation in stream-dwelling amphibian species across different elevational zones. Our results revealed significant differentiation in functional trait space between low- and high-elevation groups only for *Q. Boulengeri*. Specifically, individuals occurring at higher elevations tended to possess larger heads, consistent with previous studies suggesting that high-elevation distributed amphibians may exhibit broader trophic niches [27]. Moreover, these individuals exhibited shorter and more robust forelimbs and hindlimbs, traits typically associated with enhanced locomotor performance. It is widely recognized that a robust body morphology is generally recognized as contributing to improved energy turnover and greater consumption efficiency [28]. Importantly, these observed morphological shifts align with the pronounced environmental gradient identified in our microhabitat PCA (with PC1 dominated by elevation, temperature, water body size, flow rate, and canopy density; Table 3). Based on previous studies showing that lower air and water temperatures were typically detected in high-elevation environments [18], and corroborated by our microhabitat PCA, such morphological adaptations may help individuals maintain metabolic function by enhancing heat conservation [29,30,31]. Meanwhile, these traits also contributed to a reduced volume-to-surface ratios, thereby minimizing heat loss. In contrast, no statistically significant trait differences were detected between elevational groups for *A. sinensis* and *O. margaratae* based on PERMANOVA analyses. These results suggest that these two species may exhibit limited elevational variation in morphology or that the variation is insufficient to be statistically detected. Interestingly, all three target species showed significant differences in functional traits between sexes and among seasons. This finding aligns with prior research highlighting sexual dimorphism and seasonal variation as key drivers of external morphological traits divergence in frogs [32,33]. Seasonal morphological shifts may further reflect growth-related plasticity, necessitating future studies to disentangle ontogenetic and environmental effects.

Importantly, we found that the three target species exhibited distinct distribution preferences, as visualized in the microhabitat PCA space (Figure S1). Specifically, *Q. Boulengeri* males were associated with stream segments characterized by higher temperatures, faster flow rates, larger water bodies, and lower canopy density. In contrast, *Q. Boulengeri* females were more commonly found in areas with lower temperatures, narrower water bodies, slower flow rates, and lower rock cover, as these conditions can benefit females’ breeding [34]. These intraspecific differences in habitat selection, captured by the PCA, likely contribute to the observed sex-based functional trait variations detected by PERMANOVA. Similar intraspecific differences in habitat preference have been documented in other amphibian species, such as *Andrias davidianus* and are often linked to the distinct ecological roles played by males and females within populations [35]. A comparable pattern was observed in *O. margaratae*, where males and females also occupied different microhabitats. In contrast, both sexes of *A. sinensis* tended to prefer similar microhabitats (large water bodies with high flow rates, associated with specific positions in the PCA space), which aligns with the absence of strong elevational group differences in functional traits for this species (PERMANOVA). One limitation of this study is that we did not test for interactions between elevation, sex, and season, as insufficient numbers of males or females were encountered in some transects during the sampling events. Future studies should address this by explicitly testing the interactive effects of these factors to better understand their combined influence on trait variation and habitat use.

The results of the RDA analyses indicated that the functional traits of the three target amphibian species were shaped by distinct microhabitat variables. Crucially, these findings provide mechanistic insights into the patterns observed in both the functional trait (PERMANOVA) and habitat distribution (PCA) analyses. For *Q. boulengeri*, individuals found in transects characterized by higher air humidity, flow rate, and lower rock coverage exhibited smaller heads, larger eyes, shorter forelimbs, and longer hindlimbs. These trait patterns help explain the functional trait variation observed across sexes and seasons in the PERMANOVA, as microhabitat preferences differed between males and females. Given that *Q. boulengeri* primarily inhabits and forages in aquatic environments [34], such morphological traits likely enhance their ability to detect and capture prey under fast-flowing conditions, as well as support effective locomotion such as jumping and swimming. For *A. sinensis*, water flow rate emerged as a key factor shaping traits related to locomotion (e.g., elongated limbs). Although PERMANOVA did not detect significant trait difference between elevational groups, the RDA clearly showed that flow rate is a strong driver of intraspecific trait variation in this species, acting in concert with sex and seasonal effects. These results align with previous studies that have shown the adaptation of amphibians’ traits related to swimming performance to different water flow rates [36]. Overall, the RDA results suggested intraspecific variation in functional traits across different sex and seasonal groups. This variation may stem from sexual dimorphism and the seasonal fluctuations in population structure [32], reinforcing the complex interplay between microhabitat factors (RDA), individual level attributes (sex and season), and functional morphology (PERMANOVA/PCA), highlighting the need to account for sex and developmental stage in future investigations of functional trait variations.

## 5. Conclusions

In conclusion, the present study investigated eco-morphological functional trait variation in stream-dwelling amphibian species and suggested clear associations between microhabitat conditions and morphological traits. The observed phenotypic responses to environmental variation are consistent with findings from other aquatic taxa, such as fish, and may reflect micro-evolutionary processes associated with local adaptation. These results highlight the critical role of fine-scale environmental heterogeneity in shaping functional trait expression in mountain stream amphibians, as demonstrated by our multi-analytical approach. Future studies could focus on how functional traits variation can influence amphibians’ performance, fitness, and contributions to broader ecosystem functions, especially in the context of ongoing environmental change.

## Figures and Tables

**Table 1:** Eleven eco-morphological functional traits used in this study. The letter in brackets indicates the function associated with each trait (F, food acquisition, and L, locomotion).

Functional Traits	Code	Measure	Ecological Meaning
Mass (F/L)	M	Log (M + 1)	Volume, muscle mass
Relative head length (F)	RHL	HL/SVL	Predatory and feeding capabilities
Relative head width (F)	RHW	HW/SVL	Predatory and feeding capabilities
Relative snout length (F)	RSL	SL/SVL	Predatory and feeding capabilities
Relative diameter of eye (F)	RED	ED/SVL	Prey detection and discovery of natural predators
Relative interorbital space (F)	RIOS	IOS/SVL	Prey detection and discovery of natural predators
Relative length of lower arm (L)	RLAL	LAL/SVL	Jumping, locomotion performance
Relative length of hand (L)	RLH	LH/SVL	Jumping, locomotion performance
Relative hindlimb length (L)	RHLL	HLL/SVL	Swimming style, digging nests, and jumping
Relative tibia length (L)	RTL	TL/SVL	Swimming style, digging nests, and jumping
Relative width of tibia (L)	RTW	WT/SVL	Swimming style, digging nests, and jumping

**Table 2:** Pearson correlation coefficients between the two principal component axes and the twelve functional traits for the three species. Significant *p* values are in bold.

Functional Traits	*Q. boulengeri*		*A. sinensis*		*O. margaratae*	
	PC1 (28.58%)	PC2 (16.74%)	PC1 (41.52%)	PC2 (12.66%)	PC1 (32.55%)	PC2 (18.50%)
M	−0.53	−0.16	0.84	−0.84	0.92	−0.24
RHL	0.31	**1.02**	**−** **0.** **94**	−0.16	−0.92	−0.40
RHW	**1** **.** **17**	0.35	**−** **1** **.** **41**	0.05	**−** **1.31**	0.02
RSL	0.88	0.37	**−** **1** **.** **14**	0.35	**−** **1.** **22**	−0.46
RED	0.93	−0.10	−0.91	−0.40	−0.73	−0.57
RIOS	0.24	0.95	−0.81	0.10	−0.73	−0.36
RLAL	−0.27	0.92	−0.40	0.40	−0.87	0.46
RLH	**1.10**	0.22	**−** **1** **.** **27**	0.25	**−** **1.22**	−0.11
RHLL	**1.** **01**	**−** **0.** **89**	**−** **1** **.** **20**	−0.56	−0.56	**1.** **41**
RTL	**1.04**	−0.38	**−** **1** **.** **23**	−0.09	−0.88	**1.09**
RTW	−0.38	−0.09	−0.31	**−** **1** **.** **36**	0.60	**1.10**

**Table 3:** Pearson correlation coefficients between the two principal component axes and the nine microhabitat variables for the three species. Significant *p* values are in bold.

Microhabitat Variables	PC1 (49.85%)	PC2 (17.95%)
AT	−1.67	−0.76
AH	0.59	0.60
Wt	**−** **1** **.89**	0.03
pH_w	0.68	0.75
Wd	**−** **1.81**	−0.57
Ww	**−** **1.51**	**1.36**
Cd	**1** **.78**	0.61
Rc	0.36	**−** **1.78**
Fr	**−1.49**	**0.** **90**
Ele	**2.03**	−0.08

## Data Availability

The datasets presented in this study are available from the corresponding author on reasonable request.

## Supplementary Materials

The following supporting information can be downloaded at https://www.mdpi.com/article/10.3390/ani15152243/s1: Table S1: Details of the ten transects. Table S2: Eco-morphological functional traits of the three target species at different elevations. Figure S1: Distributions of amphibians in the environmental PCA space.
