# Supplementary material for: Intraspecific Variations in Ecomorphological Functional Traits of Montane Stream-Dwelling Frogs Were Driven by Their Microhabitat Conditions
Source: Animals (Basel). 2025 Jul 30;15(15):2243. doi: 10.3390/ani15152243 (PMC12345442; doi:10.3390/ani15152243)
Supplement: Supplementary file 1 [file animals-15-02243-s001.zip › animals-3569938-supplementary.pdf]

Table S1 Details of the ten transects.

Tansects	Length (m)	Elevation (m)	Longitude (E°)	Latitude (N°)
Shuitianba	200	467	110.0433	29.6686
Dujiazui	200	475	110.0664	29.6891
Nanmuping	200	520	110.0478	29.7419
Chewan	200	778	110.0553	29.7594
Gongtongwan	200	965	110.0916	29.7894
Sanbaizha	200	1068	110.0564	29.7685
Miaowan	200	1188	110.0672	29.7731
Daping	200	1300	110.1111	29.7992
Yangjiangping	220	1429	110.0944	29.7860
Futianya	200	1492	110.1214	29.7878

Table S2. Eco-morphological functional traits of the three target species at different elevations.

Species	Group	Sample size	M	RHL	RHW	RSL	RED	RIOS	RLAL	RLH	RHLL	RTL	RTW
Q. boulengeri	L	24	**1.75± (0.010)**	0.36± (0.001)	0.44± (0.002)	0.17± (0.003)	0.10± (0.001)	0.07± (0.001)	0.20± (0.002)	0.23± (0.001)	**1.60± (0.110)**	0.48± (0.005)	0.16± (0.001)
	H	31	**1.91± (0.009)**	0.35± (0.002)	0.43± (0.001)	0.16± (0.002)	0.09± (0.001)	0.08± (0.001)	0.20± (0.002)	0.23± (0.001)	**1.51± (0.006)**	0.48± (0.001)	0.17± (0.001)
A.sinensis	L	40	1.16± (0.004)	0.32± (0.001)	0.35± (0.001)	0.18± (0.002)	0.10± (0.001)	0.09± (0.001)	0.19± (0.001)	0.24± (0.001)	1.62± (0.003)	0.49± (0.001)	0.14± (0.001)
	H	27	1.17± (0.004)	0.32± (0.001)	0.35± (0.001)	0.19± (0.002)	0.10± (0.001)	0.09± (0.001)	0.19± (0.001)	0.26± (0.001)	1.62± (0.005)	0.50± (0.002)	0.15± (0.001)
O.margaratae	L	25	1.64± (0.006)	0.31± (0.001)	**0.29± (0.001)**	0.12± (0.001)	0.09± (0.001)	0.08± (0.001)	0.20± (0.001)	0.23± (0.001)	1.74± (0.003)	0.53± (0.001)	0.15± (0.001)
	H	55	1.68± (0.003)	0.32± (0.001)	**0.31± (0.001)**	0.13± (0.001)	0.10± (0.001)	0.08± (0.001)	0.19± (0.001)	0.23± (0.001)	1.72± (0.002)	0.53± (0.001)	0.15± (0.001)

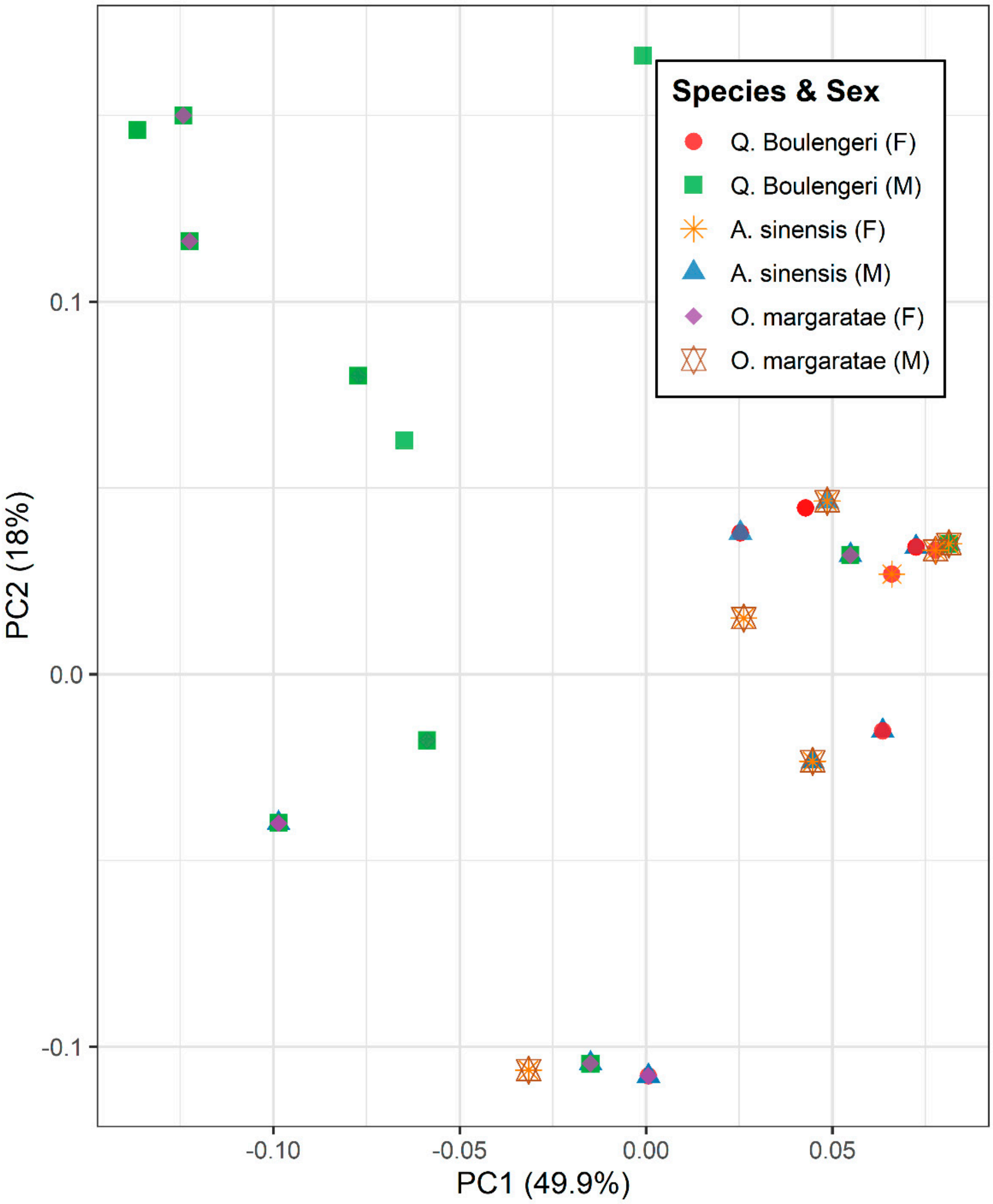

Figure S1 Distributions of amphibians in the environmental PCA space.
